# Does attending an exercise class with a spouse improve long-term exercise adherence among people aged 65 years and older: a 6-month prospective follow-up study

**DOI:** 10.1186/s12877-017-0554-9

**Published:** 2017-07-31

**Authors:** Yosuke Osuka, Songee Jung, Taeho Kim, Yoshiro Okubo, Eunbi Kim, Kiyoji Tanaka

**Affiliations:** 1 Research Team for Promoting Independence of the Elderly, Tokyo Metropolitan Institute of Gerontology, Itabashi, 173-0015 Tokyo Japan; 2 Faculty of Health and Sport Sciences, University of Tsukuba, Tsukuba, 305-8577 Ibaraki Japan; 3 The Japan Society for the Promotion of Science, Chiyoda, 102-8472 Tokyo Japan; 4 Department of Preventive Gerontology, Center for Gerontology and Social Science, National Center for Geriatrics and Gerontology, Obu, Aichi 474-8511 Japan; 5 Graduate School of Comprehensive Human Sciences, University of Tsukuba, Tsukuba, 305-8577 Ibaraki Japan; 6 Falls, Balance and Injury Research Centre, Neuroscience Research Australia, Sydney, 2031 New South Wales Australia

**Keywords:** Older married couples, Exercise adherence, Exercise social support

## Abstract

**Background:**

Family support can help older adults better adhere to exercise routine, but it remains unclear whether an exercise program targeting older married couples would have stronger effects on exercise adherence than would a program for individuals. The purpose of this study was to determine the effects of an exercise program on the exercise adherence of older married couples over a 24-week follow-up period.

**Methods:**

Thirty-four older married couples and 59 older adults participated in this study as couple and non-couple groups (CG and NCG, respectively). All participants attended an 8-week supervised program (once a week and a home-based exercise program comprising walking and strength exercises) and then participated in a follow-up measurement (24 weeks after post-intervention measurement). Exercise adherence was prospectively measured via an exercise habituation diary during the follow-up period—specifically, we asked them to record practice rates for walking (≥2 days/week) and strength exercises (≥6 items for 2 days/week). A multivariate logistic regression analysis was conducted to obtain the CG’s odds ratios (ORs) and 95% confidence intervals (CIs) for adherence to walking and strength exercise adjusted for potential confounders (with NCG as the reference).

**Results:**

Although the adherence rate of walking exercise in the CG was significantly higher than that in the NCG (29.2%; *P* < 0.001), there was no significant difference in the adherence rate of strength exercise between the two groups (*P* = 0.199). The multivariate logistic regression analysis showed that CG had significantly higher odds of adherence to walking exercise compared with the NCG (3.68 [1.57–8.60]). However, the odds of adherence to strength exercise did not significantly differ between the two groups (1.30 [0.52–3.26]).

**Conclusions:**

These results suggest that an exercise program targeting older married couples may be a useful strategy for maintaining walking adherence, even six months after the supervised program has ceased. A blinded randomized controlled trial will be needed to confirm this conclusion.

**Trial registration:**

Retrospectively registered. UMIN Clinical Trials Registry (Registered: 02/11/16) UMIN000024689.

## Background

Regular exercise is widely known as one of the most important health behaviors for maintaining quality of life and preventing falls, hospitalization and functional impairment among older adults [1]. Despite these benefits, around 50% of Japanese older adults do not participate in regular exercise [2], and approximately 50% of older adults fail to continue exercising within 6 months [3]. Therefore, strategies to maintain good exercise adherence for a long period are the key to a successful exercise program [4].

Although there are some exercise programs contrived to promote exercise adherence, such as telephone [5] and mailing [6] services, spousal-pair-based exercise programs are expected to be an innovative and gratis approach to improving exercise participation among older adults [7]. A cross-sectional study found that the husband’s physical activity level was associated with the wife’s physical activity level for both structured and unstructured physical activities [7]. Wallance et al. retrospectively compared exercise adherence over the 12-month intervention between middle-aged couples and non-couples, and reported that attendance and drop-out rates were significantly better among the couples compared to non-couples [8]. Our previous study also identified that attending an exercise class as older married couples significantly enhanced exercise adherence during a 3-month intervention period [9]. These previous studies indicate that involvement of one’s spouse may be an effective strategy to improve exercise adherence among older adults. Social support, such as support from a spouse, influenced exercise adherence via exercise self-efficacy [10]. As such, we hypothesized that individuals who participated in an exercise class with their spouse would maintain higher exercise adherence compared to individuals who participated in the program alone, since attending the exercise class with a spouse would enhance self-efficacy. However, to our knowledge, there were no prospective follow-up studies that examined the effect of spousal-based exercise programs on long-term exercise adherence among older adults.

Therefore, the purpose of this prospective follow-up study was to examine the effects of an exercise program for older married couples on long-term exercise adherence.

## Methods

### Study design and participants

We conducted a non-randomized controlled intervention between May and July 2014 with a 24-week follow-up period, which continued until January 2015. The follow-up period was set to 24 weeks because a previous study has reported that adherence to exercises learned in a program typically declines within 6 months of that program [3]. This study was conducted at the University of Tsukuba and a public community hall in Ibaraki, Japan. All participants were recruited through newspaper advertisements posted throughout the southern area of Ibaraki in March 2014. The inclusion criteria were as follows: (1) aged 65 years or older, (2) not restricted from exercising by a physician, (3) without regular exercise habits, and (4) not being classified as “assistance required” or higher according to the Japanese long-term care insurance system. In total, 95 individual older adults and 61 older married couples enrolled in the study as the non-couple group (NCG) and couple group (CG), respectively. The exclusion criteria were as follows: (1) already engaged in at least walking exercise for 150 min per week and strength exercise for 2 days per week [1] (*n* = 50) and (2) already participating in another clinical trial (*n* = 34). Six participants withdrew before the baseline measurement because of time constraints or hospitalization. The remaining 127 participants (NCG: *n* = 59, CG: *n* = 68) were included. All participants provided their written informed consent. We conducted this study in accordance with the guidelines of the Declaration of Helsinki. The study protocol was approved by the Ethics Committee of the University of Tsukuba, Japan (Tai25–108).

### Intervention

The intervention, which all participants attended, was a supervised exercise class administered once a week for 8 weeks. The class comprised 10–20 min of warming up, 50–60 min of the main exercise, and 10–20 min of a cool-down. The main exercise comprised walking and strength exercises. For the walking portion, participants were asked to walk outside for 20 min or longer. For the strength exercise portion, which was conducted in a fitness studio at the university or community center, participants performed six types of movement (squats, knee-ups, toe raises, calf raises, leg-side raises, and sit-ups), all of which were performed with their own body weight at a rather slow speed to ensure sufficient tension in the target muscle groups. They performed 15–20 repetitions of each of the six types of movement. The walking speed, repetitions and sets of strength exercise were adjusted so that an individual’s rating of perceived exertion (6 to 20) became “(13) somewhat hard” or higher [11]. All of the exercises were taught and supervised by trained instructors including a physical education teacher and a health fitness programmer. Furthermore, participants recorded in an exercise diary whether they had performed either exercise activity at home or in the exercise program on a daily basis. We recommended that the CG (1) participate and learn how to exercise in the exercise programs with their spouse, (2) practice the walking and strength exercises together in their home, and (3) keep track of each other’s exercise diaries. The NCG received an equal amount and frequency of encouragement to exercise and complete the exercise diary, but were not instructed to support each other.

### Measurements

#### Primary outcome measure

The primary outcome measure was exercise adherence during the 6-month follow-up period. Adherence to walking and strength exercises were defined as walking at least twice a week (≥20 min each time) and performing the strength exercise at least twice a week (i.e., 6 items × 2 sets = 12 sets per week), respectively [9, 12].

#### Secondary outcome measure

The secondary outcome measures were exercise-related social support and self-efficacy. Exercise-related social support was assessed using a questionnaire [13], wherein a higher score indicated greater exercise support from family and friends. We added an original item to this questionnaire that assesses support from the exercise instructor, because they supervised participants in the walking and strength exercises and checked participants’ exercise diaries during the supervised period. This questionnaire comprised 13 items, each rated on a six-point scale (0 = does not apply, 1 = none, 2 = rarely, 3 = sometimes, 4 = often, 5 = very often). Participants answered all 13 items for each of the three supporters (family, friends, and exercise instructors). The scores of the 13 items for each supporter were then summed (for a total range of 0–65). Exercise self-efficacy (i.e., ability to overcome various barriers to continue practicing exercise) was measured using the five-item Exercise Self-Efficacy Scale [14]. This scale assessed an individual’s degree of confidence in practicing regular exercise under different conditions. Each item began with the stem, “I am confident that I can practice regular exercise when…” (e.g., “I am tired”), and was rated on a scale ranging from 1 (not at all confident) to 5 (very confident). These scores were then averaged. Age, sex, family composition, medication use, medical conditions (i.e., history of stroke, hypertension, diabetes mellitus, heart disease, respiratory disease, and osteoporosis), and presence of joint pains (i.e., low back pain, hip pain, and knee pain) were measured as demographic variables. The clinical conditions and joint pains were determined based on self-report questionnaires.

#### Sample size

The sample size was calculated to detect a 20-point difference in the adherence rate of walking and strength exercises between the two groups during the follow-up period. To detect such a difference, we set the α-error and power to 0.05 and 80%, respectively. The sample size calculation revealed that 62 participants were needed for each group.

### Statistical analysis

Missing data were replaced by the last observation carried forward. Student *t*-tests for continuous variables and chi-square tests for categorical variables were used to identify differences in the baseline characteristics between the two groups. A chi-square test was also used to identify group differences in the adherence rates of walking and strength exercise throughout the follow-up period. We conducted a multivariate logistic regression analysis, while adjusting for all variables that significantly differed between the two groups at baseline, to obtain the CG’s odds ratios (ORs) and 95% confidence intervals (CIs) for the adherence to walking and strength exercise. The NCG was used as the reference in this regression analysis.

To identify group differences in the adherence rates of walking and strength exercise throughout the intervention and follow-up periods, we applied Student’s t-tests for the frequency of each exercise and a chi-square test for the rates of walking and strength exercise participation. We then conducted an analysis of covariance (ANCOVA) while adjusting for variables that significantly different between the two groups at baseline to identify changes in the patterns of exercise social support and exercise self-efficacy during the intervention and follow-up periods between the two groups. All of the analyses were performed using IBM SPSS Statistics 22.0 (IBM Corp., Armonk, NY, USA). A *P* < 0.05 was considered significant.

## Results

### Study flow

**Fig. 1:**
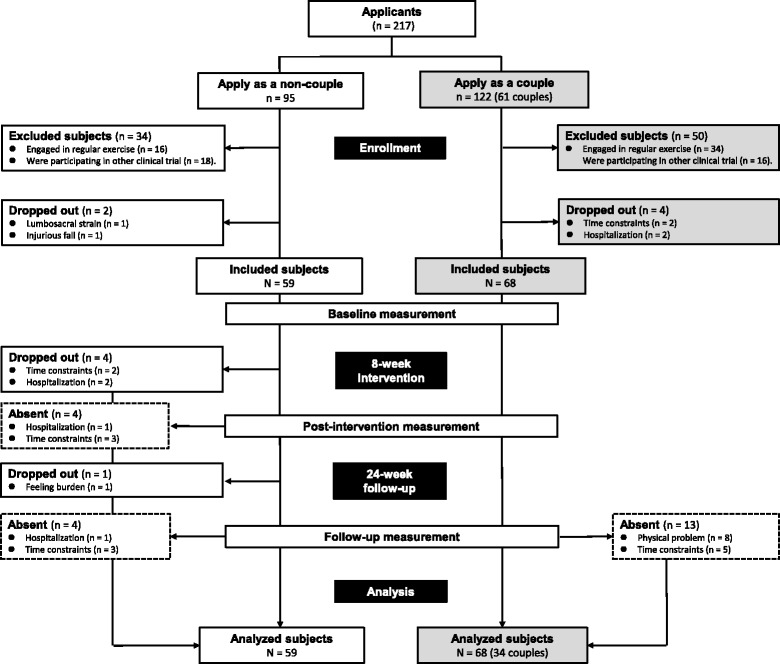
Flowchart of the study participants. Note: Data are shown as means and standard deviations

Figure 1 shows the flow of participation in the study. Thirty-four older married couples and 59 older adults were enrolled in this study as members of the CG and NCG, respectively. Five participants in the NCG dropped out because of time constraints (*n* = 2) and hospitalization (*n* = 2) during the intervention period, or because of feeling burdened (*n* = 1) during the follow-up period. Another four participants in the NCG dropped out at the post-intervention measurement because of hospitalization (*n* = 1) and time constraints (*n* = 3). Finally, four participants in the NCG and 13 participants in the CG dropped out at the follow-up measurement because of hospitalization (*n* = 1), physical problems (*n* = 8), and time constraints (*n* = 8).

### Baseline analysis

Table 1 summarizes the characteristics of the study participants at baseline. A Student’s *t-*test and a chi-square test indicated that the two groups differed significantly in terms of age, gender, cohabitee (spouse, child, and grandchild), living alone, and exercise social support from family and friends.

**Table 1:** Baseline characteristics of the study participants

	Non-couples *n* = 59	Couples *n* = 68	*P*
Age, year	71.9 ± 5.2	69.5 ± 3.8	0.003
Gender, men/women	11/48	34/34	< 0.001
Cohabitee, n (%)			
Spouse	38 (64.4)	68 (100.0)	< 0.001
Child	15 (25.4)	33 (48.5)	0.007
Grandchild	3 (5.1)	14 (20.6)	0.010
Living alone, n (%)	12 (20.3)	0 (0)	< 0.001
Medical history, n (%)			
Hypertension	20 (33.9)	28 (41.2)	0.399
Diabetes	7 (11.9)	9 (13.2)	0.816
Heat disease	6 (10.2)	3 (4.4)	0.207
Respiratory disease	4 (6.8)	2 (2.9)	0.309
Osteoporosis	1 (1.7)	5 (7.4)	0.134
Hyperlipidemia	11 (18.6)	10 (14.7)	0.551
Osteoarthritis	7 (11.9)	3 (4.4)	0.120
Joint pain, n (%)			
Low back pain	11 (18.6)	14 (20.6)	0.783
Shoulder pain	13 (22.0)	8 (11.8)	0.120
Hip pain	7 (11.9)	2 (2.9)	0.051
Knee pain	14 (23.7)	13 (19.1)	0.526
Social support score			
Family	29.8 ± 10.5	33.8 ± 10.7	0.035
Friends	30.7 ± 10.6	24.8 ± 10.5	0.002
Exercise instructor	24.3 ± 11.3	22.6 ± 12.0	0.419

### Exercise adherence

**Fig. 2:**
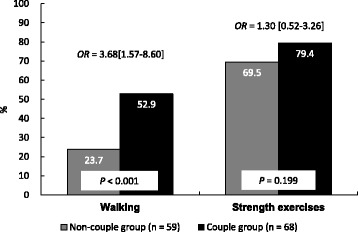
Comparisons of exercise adherence between the couple and non-couple groups during the follow-up period. Note: OR: odds ratio. ORs were adjusted for age and gender. *P* values were calculated using a chi-squared test

Figure 2 shows the results for the comparison of exercise adherence during the follow-up period between the two groups. Although the adherence rate of walking exercise in the CG was significantly higher than that in the NCG (CG: 23.7%, NCG: 52.9%, *P* < 0.001), the adherence rate in the strength exercises did not differ between the two groups (CG: 79.4%, NCG: 69.5%, *P* = 0.199). The logistic regression analysis showed that the CG had significantly greater odds of the adherence to walking exercise compared with the NCG (OR 3.68, 95% CI [1.57–8.60]). However, the odds for the adherence to strength exercise did not differ between the two groups (OR 1.30, 95% CI [0.52–3.26]).

**Fig. 3:**
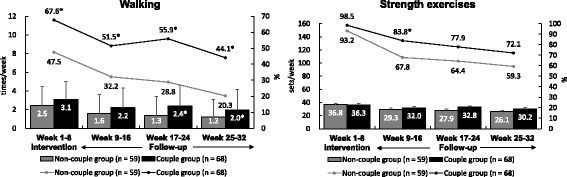
Comparisons of exercise adherence between the couple and non-couple groups during the exercise intervention and follow-up period. Note: Line graph indicates the rate of exercise participation. The bar graph indicates the means and standard deviations of exercise frequency. *: *P* < 0.05 (comparison with the non-couple group)

Figure 3 shows a comparison of exercise adherence during the intervention and follow-up periods between the two groups. The adherence rate of walking exercise in the CG was significantly higher than that in the NCG during the intervention and follow-up periods. Although the adherence rate of strength exercise was significantly higher in the CG compared with the NCG between weeks 9 and 16, there were no significant differences in these rates during weeks 17–24 and 25–32.

### Relation of exercise adherence to social support and self-efficacy

**Fig. 4:**
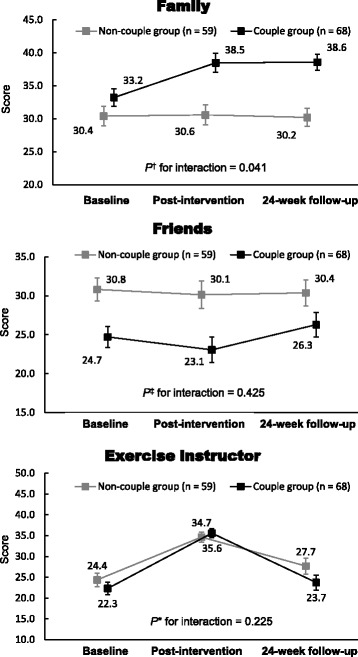
Comparisons in change patterns in exercise social support between the couple and non-couple groups during the exercise intervention and follow-up period. Note: Data are shown as estimated means and standard errors. ^†^: *P* values were calculated using an analysis of covariance adjusted for age, sex, and exercise social support from family at baseline. ^‡^: *P* values were calculated using an analysis of covariance adjusted for age, sex, and exercise social support from friends at baseline. *: *P* values were calculated using an analysis of covariance adjusted for age and sex

Figure 4 shows the results of the comparison of change patterns in exercise social support during the intervention and follow-up periods between the two groups. The ANCOVA showed a significant interaction of time × group for the exercise social support score for family members (*P* = 0.041). There were non-significant interactions for the scores of support from friends and exercise instructors.

Table 2 shows the results of the between-group comparison of the change patterns in exercise self-efficacy during the intervention and follow-up periods. We observed no significant interaction in exercise self-efficacy between the groups.

**Table 2:** Comparisons of change pattern in exercise self-efficacy between the couple and non-couple groups during the exercise intervention and follow-up period

	Non-couples *n* = 59	Couples *n* = 68	*P* for interaction
Extreme fatigue			
Baseline	2.0 ± 0.8	2.1 ± 1.0	0.727
Post-intervention	2.2 ± 1.1	2.4 ± 0.9	
Follow-up	2.1 ± 0.9	2.3 ± 0.9	
Bad mood			
Baseline	2.4 ± 0.9	2.4 ± 1.1	0.763
Post-intervention	2.6 ± 0.9	2.7 ± 1.0	
Follow-up	2.3 ± 0.8^b^	2.5 ± 0.9	
Busy situation			
Baseline	1.9 ± 0.9	2.1 ± 1.0	0.110
Post-intervention	2.3 ± 1.0^a^	2.3 ± 0.9	
Follow-up	2.0 ± 0.9	2.1 ± 1.0	
During vacation and traveling			
Baseline	2.7 ± 1.0	2.8 ± 1.0	0.185
Post-intervention	2.7 ± 1.1	3.4 ± 1.0^a^	
Follow-up	2.6 ± 1.0	2.9 ± 1.1^b^	
Rainy or snowy day			
Baseline	1.6 ± 0.8	1.7 ± 1.0	0.922
Post-intervention	2.2 ± 1.1^a^	2.4 ± 1.1^a^	
Follow-up	1.9 ± 1.0 ^b^	2.1 ± 1.0^a^	
Total score			
Baseline	2.1 ± 0.6	2.2 ± 0.8	0.822
Post-intervention	2.4 ± 0.8^a^	2.6 ± 0.7^a^	
Follow-up	2.2 ± 0.8	2.4 ± 0.8^b^	

Note: ^a^significant difference from baseline, ^b^significantly difference from post-intervention (*P* < 0.05)

## Discussion

To our knowledge, this study is the first to prospectively examine the efficacy of an exercise program for older married couples in maintaining exercise adherence over a 24-week follow-up period. The major finding of this study was that older couples—who were recruited, attended the exercise sessions, and exercised together—were more likely to maintain greater adherence to walking exercise over the 24 weeks following termination of the exercise program than were non-couples. We also found that the older couples received significantly greater social support from family members than did the non-couples, which might have led to their greater adherence to walking exercise after support from exercise instructors ceased.

Several recent studies have provided evidence of significant relationships between marital status and health outcomes [15–21]. Most of these studies indicated that being single, divorced, or widowed is a risk of adverse health outcomes, which suggest that the presence of a spouse is key for helping individuals adopt a more active lifestyle. Wankel et al. similarly reported that social support from a spouse can contribute to greater activity, especially among the older population [22]. Sallis et al. noted that the physical activity level of older women was more strongly associated with spousal support than was that of younger women [23]. Given the fact that support from a spouse has such a robust influence on individuals’ health behavior, especially among older adults, our study was right to confirm the efficacy of an exercise program for older married couples on exercise adherence.

The results of the current study broaden current evidence by showing that an exercise program targeting older married couples can improve regular walking exercise during both the supervised and unsupervised periods. This expands on Wallance et al.’s study, wherein they retrospectively compared the attendance and dropout rates of an exercise program during a 12-month intervention period between married pairs (16 pairs) and married singles (*n* = 32) in middle-aged adults [8]. Notably, monthly attendance was significantly higher and dropout rate was significantly lower (by 13.9% and 36.7%, respectively) among married pairs than among married singles. Interestingly, the most frequent reason for dropping out was lack of support from the spouse. Our previous study also compared the rate of full attendance for exercise programs between a CG and an NCG during an exercise intervention period [9]. This previous study showed that the full attendance rate of the CG was significantly higher (by about 9.1%) than was that of the NCG. This was particularly true in the latter half of the intervention (weeks 6–8). Additionally, the adherence rate of walking exercise in the CG was significantly higher than that of the NCG during the intervention period. Therefore, our previous study was consistent with the notion that an exercise intervention targeting older married couples may be useful for maintaining exercise program participation and walking during the intervention period.

Although the mechanism behind our results is somewhat unclear, they might be partially explainable by exercise social support. Specifically, support from spouses appears to encourage individuals to participate in walking. Oka et al. identified a number of psychological, social, and environmental factors related to exercise participation among 1932 Japanese adults using structural equation modeling [10]. They presented that social support influenced exercise practice via exercise self-efficacy. As such, a possible mechanism for the effectiveness of the intervention is that support from the spouse promotes individuals’ exercise self-efficacy, which in turn increases their motivation to participate in walking. However, we found that, although there was a significant time × group interaction for support from family (Fig. 3), there was no significant interaction for exercise self-efficacy between the two groups (Table 2). Thus, the mechanism behind our results appears to be that support from the spouse directly influences older adults’ exercise adherence, independent of exercise self-efficacy. It must be noted that we did not directly investigate the specific effect of support from a spouse; as such, the idea that the CG may have received greater support from their spouse (e.g., encouragement and motivation for walking) compared with the NCG remains mere speculation.

We found no significant difference in the adherence rate of strength exercise between the two groups. In general, exercise adherence is associated with a variety of factors that fall into a range of categories, such as routine-related, intrinsic, biophysical, psychosocial, environmental, and resource-related factors. McArthur et al. qualitatively identified the enablers and barriers to adherence to regular exercise among middle-aged women [24]. The most oft-reported enabling factor was “an established daily structure that incorporated exercise” (broad theme: routine-related factors). For example, participants who wanted to integrate regular exercise into their daily routine thought it important to make it a habitual lifestyle activity that was as unconscious as daily tooth-brushing. The frequency of strength exercise was notably higher than was that of walking, suggesting that most participants felt that strength exercise was one of their habitual lifestyle activities. As such, because most participants had integrated strength exercise into their routine lifestyle, support from a spouse might not have had any effect on the practice of this exercise. The strength exercise utilized in the present study may thus be a useful intervention for older adults who live alone, as it appears to be easy to maintain and does not require social support.

Although the adherence rate of walking exercise was significantly higher in the CG than in the NCG, both rates equally declined over time, and there was no significant interaction in walking adherence between the two groups (data not shown). Chogahara et al. reviewed various studies on how social support related to participation in exercise among older adults [25, 26]. They found that most previous studies had overly emphasized the positive aspects of social support, while its negative aspects were largely neglected. Indeed, Barnett et al. explored and described how spousal support can influence both spouses’ physical activity behavior using a qualitative approach, and suggested that excessive demands from the spouse can negatively influence adherence [27]. This means that we cannot exclude the possibility that support from spouse negatively affected exercise participation during the study period. For example, if a husband complained to his wife about her walking speed (or vice versa), it could decrease both of their motivations to continue walking. Coexistence of the negative and positive effects of spousal communication may be the reason that there was no significant interaction in walking adherence between the two groups. An educational exercise intervention that improves the effects of spousal support on exercise participation will be needed in the future for older married adults.

### Limitations

The strengths of the current study were that it was the first to prospectively identify the effects of an exercise intervention for older married couples on exercise adherence. This novel strategy has the potential to encourage older males and people with little interest in the adherence to walking exercise in health promotion activities through spousal invitation. However, there were several limitations in this study. First, there was a possibility of selection bias because participants who were interested in practicing exercise are more likely to participate in such studies. Thus, the results of this study might not be generalizable to the overall Japanese population, especially among older adults who are not interested in practicing exercise. Second, there is a possibility of arbitrary bias due to the non-randomized group allocation and because blinding was not possible in this study design. A better designed approach to stimulate exercise adherence that would allow for recruitment of control groups should be used in the future [28, 29]. These methods would provide more detailed insights into the processes underlying spousal support. Third, although the current study prospectively assessed exercise adherence using an exercise diary, which could stimulate adherence to exercise, using an objective evaluation of exercise (e.g., an accelerometer) would have provided more reliable results. Finally, the final sample size in the NCG (*n* = 59) was slightly below the required sample size (*n* = 62) because of exclusion of many participants with regular exercise habits, withdrawals after consent, and the limited study period given by our research funding. However, this study was sufficiently powered to detect the difference in adherence to walking exercise. A well-designed randomized controlled trial using an objective measurement of exercise adherence and an appropriate sample size will be needed to validate the novel findings of this study. Additionally, future studies should identify whether not-married couple units, such as pairs of friends (friend-pair-based exercise programs), have a similar effect on exercise adherence as married couples do.

## Conclusions

The practice rate of walking exercise in the CG was significantly higher than that in the NCG. The significant interaction in the exercise social support score from family indicates that support from a spouse is the key factor to promote habitual exercise, particularly walking exercise among older married couples. These results suggest the possibility of a novel strategy for older married couples that can help promote adherence to walking exercise even after the exercise class has ended.

## Abbreviations

CG: Couple group
NCG: Non-couple group
